# S100B Serum Levels Predict Treatment Response in Patients with Melancholic Depression

**DOI:** 10.1093/ijnp/pyv103

**Published:** 2015-09-12

**Authors:** Oliver Ambrée, Veerle Bergink, Laura Grosse, Judith Alferink, Hemmo A. Drexhage, Matthias Rothermundt, Volker Arolt, Tom K. Birkenhäger

**Affiliations:** Department of Psychiatry, University of Münster, Germany (Dr Ambrée, Ms Grosse, Dr Alferink, Dr Rothermundt, and Dr Arolt); Department of Psychiatry, Erasmus Medical Center Rotterdam, The Netherlands (Drs Bergink and Birkenhäger); Radiology Morphological Solutions, Rotterdam, The Netherlands (Ms Grosse); Department of Immunology, Erasmus Medical Center Rotterdam, The Netherlands (Dr Drexhage); Cluster of Excellence EXC 1003, Cells in Motion, Münster, Germany (Dr Alferink); Department of Psychiatry, St. Rochus-Hospital, Telgte, Oberhausen, Germany (Dr Rothermundt).

**Keywords:** Major depression, predictive biomarker, antidepressant response, treatment resistance, neurotrophic factor

## Abstract

**Background:**

There is an ongoing search for biomarkers in psychiatry, for example, as diagnostic tools or predictors of treatment response. The neurotrophic factor S100 calcium binding protein B (S100B) has been discussed as a possible predictor of antidepressant response in patients with major depression, but also as a possible biomarker of an acute depressive state. The aim of the present study was to study the association of serum S100B levels with antidepressant treatment response and depression severity in melancholically depressed inpatients.

**Methods:**

After a wash-out period of 1 week, 40 inpatients with melancholic depression were treated with either venlafaxine or imipramine. S100B levels and Hamilton Depression Rating Scale (HAM-D) scores were assessed at baseline, after 7 weeks of treatment, and after 6 months.

**Results:**

Patients with high S100B levels at baseline showed a markedly better treatment response defined as relative reduction in HAM-D scores than those with low baseline S100B levels after 7 weeks (*P*=.002) and 6 months (*P*=.003). In linear regression models, S100B was a significant predictor for treatment response at both time points. It is of interest to note that nonresponders were detected with a predictive value of 85% and a false negative rate of 7.5%. S100B levels were not associated with depression severity and did not change with clinical improvement.

**Conclusions:**

Low S100B levels predict nonresponse to venlafaxine and imipramine with high precision. Future studies have to show which treatments are effective in patients with low levels of S100B so that this biomarker will help to reduce patients’ burden of nonresponding to frequently used antidepressants.

## Introduction

Biomarkers are objective measures of biological processes and can be used as either a diagnostic tool to identify patients with a particular psychiatric disorder or as predictors of a patient’s response to treatment (Biomarkers Definitions Working Group, 2001). Such biomarkers, for instance, can be alterations in blood levels of single molecules, genetic variants, epigenetic changes, or neuroimaging findings, among others (Kennedy et al., 2012; Boksa, 2013). In psychiatry, there is an unmet need for biomarkers in order to reduce patients’ suffering and costs of treatment by improving diagnostics and predicting treatment response. In addition, funding agencies and pharmaceutical companies increasingly request objective measures besides clinical data to invest in psychiatric research in the future (Kapur et al., 2012).

One potential biomarker that seems to be involved in several psychiatric disorders is the S100 calcium binding protein B (S100B) (Schroeter et al., 2013; Yelmo-Cruz et al., 2013). In the brain, this protein is mainly expressed in astrocytes and to a certain extent also in oligodendrocytes (Steiner et al., 2007; Gos et al., 2013). It has several intra- and extracellular functions, including regulation of cell proliferation, differentiation and survival, and Ca^2+^ homeostasis (Rothermundt et al., 2003; Donato et al., 2009). In particular, it has been shown to exert neurotrophic functions in a dose-dependent manner stimulating neurite outgrowth, enhancing survival of neurons and supporting the development of serotonergic neurons (Alexanian and Bamburg, 1999; Huttunen et al., 2000; Eriksen and Druse, 2001).

In psychiatric disorders, S100B was investigated by assessing serum levels, levels in cerebrospinal fluid (CSF) and variants in the *S100B* gene. Genetic variants were found to be associated with the psychotic subtype of bipolar disorder, visuospatial disability in schizophrenia, and recurrence of major depression (Roche et al., 2007; Yang et al., 2009; Zhai et al., 2011). CSF levels were elevated in patients with major depression and schizophrenia compared with controls (Grabe et al., 2001; Rothermundt et al., 2004; Steiner et al., 2006). S100B levels were also found to be higher in serum samples of patients with schizophrenia, major depression, and bipolar disorder (Schroeter et al., 2013; Yelmo-Cruz et al., 2013). Nonetheless, there are some inconclusive reports whether S100B levels in major depression are always elevated (Jang et al., 2008) and if these levels decline with successful treatment (eg, Schroeter et al., 2008).

Other studies reported that elevated S100B levels in depressive patients at the beginning of an antidepressant treatment correlate with subsequent treatment response (Arolt et al., 2003; Jang et al., 2008), suggesting that elevated S100B levels might contribute to a successful treatment. Patients in these studies were treated naturalistically in a standard hospital setting. One study reported an association between elevated S100B levels and illness severity (Jang et al., 2008), leaving open whether the correlation of treatment response and S100B levels was only a statistical effect, because the more depressive patients with higher S100B levels had a larger range to improve.

Therefore, the aim of this study was to analyze the predictive value of serum S100B levels for antidepressant treatment response in a randomly assigned, double-blind treatment trial with either the serotonine-norepinephrine reuptake inhibitor venlafaxine or the tricyclic antidepressant imipramine. Based on the previous findings, it was hypothesized that patients with high S100B levels at baseline would respond better to antidepressant treatment. Since some studies also reported an association of S100B levels with depression severity, S100B levels were controlled for this association and monitored at baseline and after treatment in order to elucidate whether S100B levels in melancholic depression represent a trait marker for antidepressant responsiveness or a state marker for the current severity of the disorder.

## Methods

### Study Design

The analysis of S100B as biomarker for antidepressant response was designed as an add-on study to a clinical trial on the efficacy and tolerability of the tricyclic antidepressant imipramine and the serotonin-norepinephrine reuptake inhibitor venlafaxine (Vermeiden et al., 2013). These drugs were selected because they appeared to be more effective than selective serotonin reuptake inhibitors in inpatients with severe major depression. After a drug-free wash-out period of 7 days, patients were assigned to antidepressant treatment with either venlafaxine or imipramine in a randomized double-blind trial. The treatment phase lasted 7 weeks, during which depression severity was monitored weekly by the 17-item version of the Hamilton Depression Rating Scale (HAM-D). Blood sampling took place after the wash-out phase before treatment started (baseline), after the 7-week treatment period (+7wks), and at the 6-month follow-up investigation (+6mos). The first time point was elected to assure that patients receive the maximum dose of venlafaxine and the optimal dose with therapeutic plasma levels of imipramine for at least 4 weeks. The time point of 6 months was chosen to assess whether patients attaining response would show sustaining response after continuation treatment for another 4 months. The study was approved by the Medical Ethics Committee of the Erasmus Medical Center Rotterdam. The protocol was carried out in accordance with the ethical standards laid down in the Declaration of Helsinki (1964), as amended in Edinburgh (2000). All patients gave written informed consent after study procedures had been explained in detail.

### Patients

Patients suffering from major depression were consecutively recruited at the inpatient depression unit of the Department of Psychiatry of the Erasmus Medical Center Rotterdam. All depressed patients admitted to the depression unit meeting inclusion criteria and willing to participate enrolled in the original study, which comprised 85 patients diagnosed with major depression including melancholic features according to DSM-IV-TR (SCID-1) and a minimum HAM-D score of 17 (Vermeiden et al., 2013). Of those, 55 patients gave informed consent for the biomarker study. Another 15 patients were not included: 3 patients refused participation after randomization, 10 patients had major depression with psychotic features, and 2 patients lacked melancholic features. Thus, the present study comprised 40 consecutive patients with melancholic and without psychotic features. To be included in the study, patients were not allowed to meet one or more of the following criteria: incapable of understanding the information and to give informed consent; unable to read or write; bipolar I or II disorder; presence of psychotic features, schizophrenia, or other primary psychotic disorder; drug/alcohol dependence for the last 3 months; any clinically relevant renal, hepatic, endocrine, or neurologic illness; and use of steroids or nonsteroidal antiinflammatory drugs. Women were not included if they were pregnant or breastfeeding.

### Medication

After a drug-free wash-out period of 1 week, patients were randomized to either venlafaxine (mean daily dose 371mg, range 300–375mg/d) or imipramine (mean dose 206mg, range 50–450mg/d, blood level 200–300ng/mL) treatment. For details of dose adjustment, see Vermeiden et al. (2013). After 7 weeks, patients who achieved response (≥50% reduction in HAM-D scores) were kept on the same dose of the antidepressant without concomitant medication (except for 6 of 40 patients who received lorazepam <3mg/d). Most nonresponders received lithium as addition to the ongoing antidepressant treatment.

### Blood Sampling and Analysis

Blood samples were taken after the wash-out period, before treatment started (baseline), +7wks, and +6mos. Sampling took place at 8:00 am in the morning. Serum was stored at –80°C until collection of all samples was completed and shipped on dry ice to the Münster laboratory for measuring S100B levels. All samples were analyzed in the Elecsys S100 electrochemiluminescence assay in singles (Roche Diagnostics, Mannheim, Germany). The intra- and inter-assay coefficients of variation were 1.0 to 1.8% and 2.5 to 3.1%, respectively.

### Statistical Analysis

Histograms were assessed to check continuous variables for normal distribution. Age deviated strongly from normal distribution. Since it was only used as a predictor variable or covariate in the main analyses that did not require the assumption of normal distribution, no transformation was applied for age data, and nonparametric Spearman’s correlation was calculated for this variable in the pretest. Otherwise, Pearson’s correlations were calculated to assess the association between 2 continuous variables. Then *t* tests for independent data were calculated to compare 2 independent samples. To compare S100B levels at the 3 time points, repeated-measures ANOVAs were calculated. The main hypothesis of this study was that baseline S100B levels alone and together with relevant clinical parameters predict treatment response. The dependent variables, acute (+7wks) and long-term (+6mos) treatment responses, were defined as percentage decrease in HAM-D scores after treatment in relation to baseline scores. For the differentiation between responders and nonresponders, responders were defined as patients that reached a reduction of at least 50% in their HAM-D scores at +7wks. S100B serum levels at baseline (high, low), age, sex, HAM-D scores at baseline, recurrence of the disorder (no, yes), medication (venlafaxine, imipramine), and body mass index (BMI) were chosen as candidates for predictor variables of the linear regression model. Sample sizes of subgroups for gender, medication, response, and recurrence are depicted in supplementary Figure 1. Correlations were calculated between treatment response and these variables. Since BMI, sex, and the initial HAM-D scores did not correlate to treatment response, the final model comprised baseline S100B levels, type of medication, age, and recurrence. To approach this question from another point of view, a repeated-measures ANCOVA was calculated with Hamilton scores at baseline, +7wks, and +6mos as within-subject variables and S100B levels at baseline (high/low) as between-subjects variable including the other predictor variables as covariates. Effects were considered significant if *P*<.05. All analyses were calculated using SPSS software (IBM, version 22).

## Results

### S100B Levels, Antidepressants, Age, and Recurrence: Relation with Treatment Response

Patients were between 33 and 67 years of age (52.1±9.2, mean±SD), had a body weight of 41 to 98kg (71.0±13.0), a BMI between 15.4 and 33.3 (23.8±4.2), and a baseline HAM-D score of 18 to 30 (24.3±3.2) indicating moderate to severe depression. After 7 weeks of treatment, either with venlafaxine or imipramine HAM-D scores declined by 36.6±32.2% (range: -26.3 to 94.7%). At the 6-month follow-up, HAM-D scores were reduced by 37.8±34.7% (range: -28.6 to 96.2%) compared with baseline.

**Figure 1.**
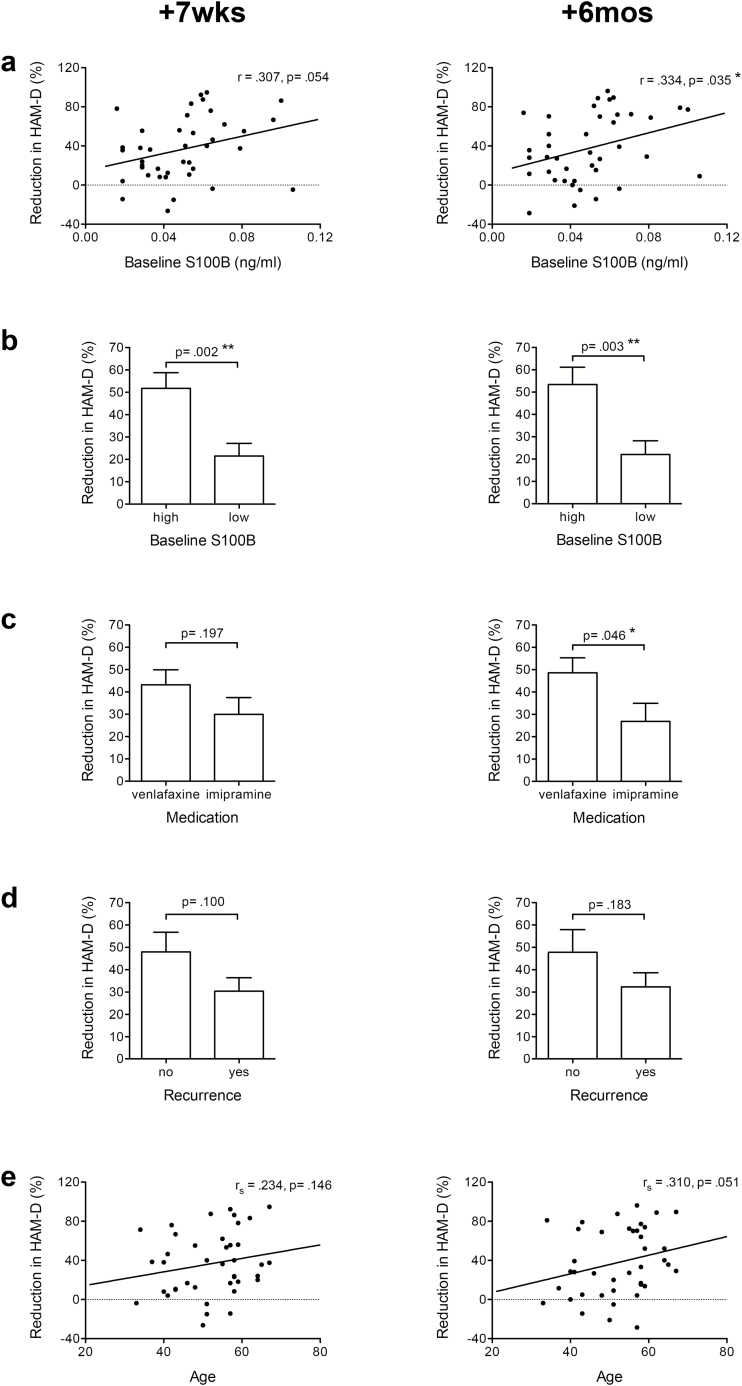
(a) There was a significant association of moderate effect size between baseline S100 calcium binding protein B (S100B) levels and treatment response at both time points after treatment. Patients with higher baseline S100B levels improved more than those with initially lower S100B levels. (b) Patients with high baseline S100B levels (≥.051ng/mL) showed a significantly larger relative reduction in Hamilton Depression Rating Scale (HAM-D) scores at both time points after treatment. (c) There was no significant difference in HAM-D reductions between venlafaxine- and imipramine-treated patients. (d) Patients with first episode depression showed a significant larger relative reduction in HAM-D scores than patients with recurrent depression after the 7-week treatment period (+7wks). (e) There was a moderate association between age and treatment response, with older patients improving a little better than younger ones at the 6-month follow-up investigation (+6mos).

First we analyzed the potential predictor variables for treatment response. Treatment response at both time points showed a moderate correlation with initial S100B serum levels at baseline (Pearson’s correlation: +7wks: *r*=.307, *P*=.054; +6mos: *r*=.334, *P*=.035) (Figure 1a). To define groups with high and low baseline levels of S100B, this variable was dichotomized by a median split. Patients with high (≥.051ng/mL) baseline S100B levels had a significantly larger improvement in HAM-D scores of about 50% compared with those with initially low levels (<.051ng/mL) showing reductions of about only 20% (*t* test: +7wks: *t*(38)=-3.350, *P*=.002; +6mos: *t*(38)=-3.172, *P*=.003) (Figure 1b).

There was no significant difference in treatment response between venlafaxine- or imipramine-treated patients at +7wks, while venlafaxine-treated patients showed a significantly better response at +6mos (*t* test: +7wks: *t*(38)=-1.314, *P*=.197; +6mos: *t*(38)=-2.063, *P*=.046) (Figure 1c). Patients with recurrent episodes had a slightly reduced but statistically not significant acute treatment response at +7wks compared with patients with a first episode of depression (*t* test: *t*(38)=-1.685, *P*=.100) while recurrence had even less influence at +6mos (*t* test: *t*(38)=-1.357, *P*=.183) (Figure 1d). Age showed a moderate positive correlation with treatment response at both time points, however, without reaching statistical significance (Spearman’s rank correlation: +7wks: *r*_*s*_=.234, *P*=.146; +6mos: *r*_*s*_=.310, *P*=.051) (Figure 1e). In contrast, there was no association between treatment response and initial HAM-D scores, gender, or BMI (supplementary Figure 2a-c). Variables that showed at least a tendency for an association with treatment response at one time point (*P*≤.1) were included in linear regression models to predict acute and long-term treatment responses.

### S100B Serum Levels Predict Treatment Response

Baseline S100B levels, medication, recurrence, and age accounted for 40.5% of the variation in the acute treatment response (adj. *R* ^*2*^=.405, *P*< .001) and for 48.5% of the long-term treatment response (adj. *R* ^*2*^=.485, *P*< .001) (Table 1a). Baseline S100B levels and medication turned out to be significant predictors for both the acute (+7wks) and long-term (+6mos) treatment response, while age was also a significant predictor for the long-term treatment response (Table 1a).

**Table 1.** (a) Multiple linear regression models of acute and long-term treatment response with predictor variables S100B levels at baseline (^a^high/low), medication (^b^venlafaxine/imipramine), age, and recurrence. (b) Simple linear regression model to predict acute and long-term treatment response from S100B serum levels at baseline as sole predictor. Bold figures indicate significant p-values and adjusted R2 values.

**a) Multiple Linear Regression Models.**	**Acute Treatment Response** **+7wks**			**Long-Term Treatment Response** **+6mos**		
	*B*	*SE*	*P*	*B*	*SE*	*P*
Baseline S100B^*a*^	38.488	8.257	<.001***	42.868	8.281	<.001***
Medication^*b*^	21.839	8.293	.013*	31.537	8.318	<.001***
Age	.800	.434	.074	1.037	.435	.023*
Recurrence (yes/no)	15.109	8.273	.076	11.904	8.298	.160
Adjusted *R* ^*2*^	**.405**			**.485**		
*F* (df)	7.645 (4,35)		<.001***	10.194 (4,35)		<.001***
**b) Simple Linear Regression Models.**	**Acute Treatment Response +7wks**			**Long-Term Treatment Response +6mos**		
	***B***	***SE***	***P***	***B***	***SE***	***P***
Baseline S100B^*a*^	30.335	9.055	.002*	31.333	9.879	.003**
Adjusted *R* ^*2*^	**.208**			**.188**		
*F* (df)	11.225 (1,38)		.002**	10.059 (1,38)		.003**

Since S100B levels have been the strongest and most significant predictor for the treatment response at both time points, we calculated simple linear regression models with S100B levels at baseline (high/low) as the only predictor. While these models were significant for both time points, the predictive values with adjusted *R* ^*2*^=.208 (+7wks, *P*=.002) and adjusted *R* ^*2*^=.188 (+6mos, *P*=.003) were rather modest (Table 1b). The analysis of the statistical quality of serum S100B as treatment predictor (Table 2) revealed a sensitivity of about 79%, specificity of 65%, positive predictive value of 55%, and number of false positives of 22.5%. However, the negative predictive value was 85%, while the false negatives were only 7.5%, indicating that patients with low S100B levels can be classified with high precision as treatment nonresponders.

**Table 2.** Calculation of sensitivity, specificity, positive/negative predictive values, and rate of false positives/negatives for S100B levels as marker of therapy response using the median concentration at baseline as cut-off value.

		**Responders**	**Nonresponders**	**Total**
Baseline S100B	≥.051 (ng/mL)	11	9	20
	<.051 (ng/mL)	3	17	20
	Total	14	26	40

HAM-D scores at baseline did not differ between patients with high and low baseline S100B levels. However, patients with high baseline S100B levels showed larger reductions in HAM-D scores after 7 weeks and 6 months than those with initially low levels (repeated-measures ANCOVA on HAM-D scores controlled for medication, age, and recurrence: time x S100B high/low: *F*_1.4,49.0_=19.203, *P*<.001) (Table 3A).

**Table 3.** (a) Hamilton scores at baseline and after 7 weeks and 6 months for all patients and separated for those with high and low baseline S100B levels. (b) S100B levels at the 3 different time points: baseline, +7wks, and +6mos in all patients as well as separated by response.

a) Hamilton Scores.									
	**All patients**			**Baseline S100B high**			**Baseline S100B low**		
	**N**	*M*	95 % CI	N	*M*	95 % CI	N	*M*	95 % CI
Baseline	40	24.28	[23.3,25.3]	20	24.85	[23.1,26.6]	20	23.70	[22.5,24.9]
+7wks	40	15.43	[12.9,17.9]	20	12.40	[8.4,16.4]	20	18.45	[15.7,21.2]
+6mos	40	15.10	[12.4,17.8]	20	11.90	[7.6,16.2]	20	18.30	[15.2,21.4]
**b) S100B Levels.**									
	**All patients**			**Responders**			**Nonresponders**		
	**N**	***M* (ng/mL)**	**95 % CI**	**N**	***M* (ng/mL)**	**95 % CI**	**N**	***M* (ng/mL)**	**95 % CI**
Baseline	40	.050	[.043,.057]	14	.061	[.047,.074]	26	.044	[.036,.052]
+7wks	39	.049	[.042,.057]	14	.063	[.049,.078]	25	.041	[.034,.049]
+6mos	20	.055	[.043,.067]	12	.064	[.045,.082]	8	.042	[.033,.051]

Please note that at +6mos, fewer blood samples were available, especially from nonresponders, probably accounting for a slightly elevated mean level in the total group at this time point.

### S100B Serum Levels Are Stable over the Course of Treatment

To control whether elevated S100B levels are associated with more severe depression and therefore are more likely to be associated with an increased treatment response, we calculated the correlation between baseline S100B levels and severity of depression at baseline as assessed by HAM-D scores. There was no significant correlation between these variables (Pearson correlation: *r*=.013, *P*=.938), indicating that elevated S100B levels are independent from depression severity. In addition, S100B levels did not change with clinical improvement over time, as indicated by the lack of a significant difference between S100B levels at the 3 time points (Table 3B) before treatment (baseline), +7wks, and +6mos, either as main effect of time (*F*_1.4,25.8_=.532, *P*=.535) or as interaction effect between time and treatment response (*F*_1.4,25.8_=.128, *P*=.812). However, as could be expected by the predictive property of S100B levels, responders had significantly higher S100B levels than nonresponders (*F*_1,18_=5.635, *P*=.029). Since the number of samples for the final time point +6mos was quite small, this repeated-measures ANOVA was also calculated for the first 2 time points only, revealing similar results (time: *F*_1,37_=.046, *P*=.832; time x response: *F*_1,37_=.810, *P*=.374; response: *F*_1,37_=8.998, *P*=.005).

## Discussion

This study examined whether circulating S100B levels in the serum of patients with melancholic depression are associated with outcome after antidepressant treatment. As hypothesized, patients with higher baseline levels of S100B showed significantly larger reductions in HAM-D scores after treatment compared with those with lower S100B baseline levels. Patients with low S100B levels could be classified as nonresponders with high precision. The severity of depression was not associated with S100B levels. Moreover, S100B levels did not differ between baseline, +7wks, and +6mos time points.

S100B has been shown to exert neurotrophic and neuroprotective effects, especially on serotonergic neurons (Alexanian and Bamburg, 1999; Huttunen et al., 2000; Eriksen and Druse, 2001), when it was available at nanomolar concentrations in contrast to neurotoxic effects at micromolar concentrations (Fano et al., 1995). On the basis of S100B levels in the lumbar cerebrospinal fluid of depressive patients, ventricular CSF levels have been estimated, strongly indicating that, in mood disorders extracellular S100B levels in the brain are in the nanomolar range far below micromolar concentrations (Schroeter et al., 2013). Since there is strong evidence for reduced neurotrophic factor expression, impaired neuroplastic function, and even mild forms of brain atrophy in certain brain structures in major depression, it is assumed that restoring neuroplastic function in depressive patients is a fundamental part of behavioral improvement (Castrén, 2013). For instance, it has been shown that the antidepressant action of fluoxetine requires the induction of adult neurogenesis (Santarelli et al., 2003). Here it is of interest to note that intracerebroventricular and even intraperitoneal application of S100B increases progenitor cell proliferation as well as neuronal differentiation and survival of newborn cells in mice after brain injury (Kleindienst et al., 2005, 2013). So, elevated levels of S100B as found in one-half of the patients could indicate an increased neurotrophic potential and thereby contribute to therapy response by increasing brain plasticity. Indeed, it has recently been shown that the action of the selective serotonin reuptake inhibitor fluoxetine involves the release of S100B from terminals of serotonergic neurons at the locus coeruleus where it increases the expression of the serotonin transporter at the site of norepinephrine synthesis (Baudry et al., 2010). Both drugs, the serotonine-norepinephrine reuptake inhibitor venlafaxine and the tricyclic antidepressant imipramine, work as inhibitors of both serotonin and norepinephrine reuptake (Holliday and Benfield, 1995; Humble, 2000) and thus might directly modulate these processes. These findings might suggest that patients with higher S100B expression have an increased availability of this neurotrophic factor, which may promote the antidepressant action at the intersection of serotonergic and noradrenergic neurotransmission. It would be of interest to know whether elevated S100B levels are especially beneficial for the successful action of certain antidepressants such as those with a strong effect on the inhibition of serotonin reuptake, while patients with low S100B levels might better benefit from different antidepressants or other treatments.

The main finding of the present study that baseline S100B levels are associated with antidepressant response is in accordance with 2 previous reports (Arolt et al., 2003; Jang et al., 2008). In addition, in the present study S100B levels were not associated with severity of depression. Instead, our findings suggest that S100B predicts treatment response independent of depression severity. In our study, S100B levels did not change over the course of treatment. While there are some inconsistent reports whether S100B levels decline with treatment response (Schroeter et al., 2002) or not (Hetzel et al., 2005; Jang et al., 2008; Schroeter et al., 2008), the present study strongly suggests that S100B rather is a trait marker for antidepressant responsiveness than a state marker for depression severity. In addition, there exist rather inconclusive findings whether S100B levels in serum or cerebrospinal fluid of depressive patients are elevated compared with healthy controls (Grabe et al., 2001; Schroeter et al., 2002, 2008; Hetzel et al., 2005) or not (Jang et al., 2008; Schmidt et al., 2015). These contradictory findings indicate that the role of S100B in major depression is rather complex and still far from being understood. In a recent study, S100B overexpressing mice displayed an increased reactivity to environmental stimuli as shown by an increased behavioral and neural plasticity (Buschert et al., 2013). Thus, elevated S100B expression might be associated with a differential susceptibility to environmental stimuli and could act as a plasticity factor in the way it has been proposed by Belsky and Pluess (2009). Consequently, functional variants in the S100B gene would represent plasticity alleles rather than risk alleles (Belsky et al., 2009).

Since S100B is not only expressed in the brain but also in a number of peripheral tissues such as white fat, skeletal muscle, or heart (Zimmer et al, 1995), serum levels need not necessarily reflect individual variation in S100B expression in the brain. They could also be influenced by altered extra cranial S100B expression, for example, in patients with comorbid heart disease, obesity, or metabolic syndrome (Mazzini et al., 2005, 2007, 2009; Steiner et al., 2010a, 2010b), which might be a possible explanation for the sometimes contradictory findings on the role of S100B in major depression that are found in the literature. One study showed an association of S100B levels with BMI that was mainly due to subjects with a BMI > 30 who showed markedly higher S100B levels compared with patients with a smaller BMI (Steiner et al., 2010a). In our study, BMI, in contrast to S100B levels, showed no association with treatment response, suggesting that the effects of S100B were not due to variations in BMI.

It should be noted that altered levels of S100B have been found in other neuropsychiatric disorders such as schizophrenia, autism, and Alzheimer’s disease (Peskind et al., 2001; Al-Ayadhi and Mostafa, 2012; Aleksovska et al., 2014), implicating that alterations in levels of this molecule are not specific for major depression. Altered S100B might have different causes and consequences in these disorders, and it is not clear whether its main function is always the contribution to neuroplasticity. This may be true when S100B levels are in a physiological range, as is the case in MDD patients, or during early stages of neurodegenerative diseases. However, there is also evidence that S100B might be a glial marker for neuroinflammation or even contribute to inflammation and neurodegeneration in pathological conditions (Rothermundt et al., 2003; Donato et al., 2009; Mori et al., 2010).

Despite the precise prediction of treatment nonresponse by low S100B levels, high serum S100B is not sufficient as sole predictor of antidepressant treatment. This is the same with a number of other candidate biomarkers for antidepressant treatment responses like T-lymphocytic CREB, inflammatory markers, functional or structural neuroimaging, or gene-environment interactions in the etiology of the disorder (Fu et al., 2013; Klengel and Binder, 2013; Lim et al., 2013; Raison et al., 2013; Sämann et al., 2013; Uher et al., 2014). Since S100B also has an immune-modulatory function (Donato et al., 2009) and immune dysregulation has been frequently described in major depression (eg, Miller et al., 2009; Zunszain et al., 2013; Carvalho et al., 2014; Grosse et al., 2015), it would be interesting to analyze how S100B relates to markers of the immune system. Our group has previously shown that increased percentages of CD8+ cytotoxic T cells and decreased percentages of natural killer cells are associated with a poor response to antidepressant treatment in the same cohort of patients with melancholic depression (Grosse et al., 2015). In addition, we described increased proinflammatory gene expression in these patients (Carvalho et al., 2014). Therefore, it could be a valuable approach to integrate all these parameters into a combined biosignature, analyze its predictive value for antidepressant treatment response, and test it in an independent cohort of patients. To reach the goal of sufficient prediction of treatment response, such an approach of combining multiple biomarkers and defining algorithms for multivariate analysis would probably be necessary (Kennedy et al., 2012).

### Limitations

There are several limitations to the present study. First, in this randomly controlled treatment trial, only severely depressed patients with melancholic features were included. Thus, it is not possible to draw conclusions on patients with other forms of depression or depression of different subtypes such as atypical, psychotic, anxious, or unspecified depression. Second, although the sample size of the present study was sufficient to analyze the predictive value of S100B levels for the treatment response, it was too small to allow for the comparison of the antidepressant treatment subgroups. Based on the finding that S100B acts as a mediator between serotonergic terminals and serotonin transporter expression in the noradrenergic locus coeruleus (Baudry et al., 2010), it can be hypothesized that patients with higher S100B levels might profit more from certain types of antidepressants that act preferentially on the serotonergic system from those that have different modes of action. Therefore, the choice of only two antidepressants is another limitation that does not allow the generalization of the findings to other antidepressants. Finally, it should be mentioned that serum S100B levels are only an indicator of S100B concentrations in the brain. It has been estimated that S100B is able to pass the blood brain barrier to a certain extent (Reiber et al., 2001), but it has to be kept in mind that extra-cranial sources of S100B might interfere with this interpretation. While our rather homogenous study population did not seem to be affected by this interference in a considerable extent, this situation can be different in more heterogeneous patient groups.

## Conclusion

Despite the aforementioned limitations, this study provides considerable evidence for serum S100B levels as a predictive biomarker of antidepressant treatment response in patients with melancholic depression. Especially nonresponders can be detected by low S100B levels with high precision. Since S100B serum levels did not change with successful treatment and were not associated with severity of depressive symptoms, S100B might be utilized as a trait marker for neural plasticity rather than as a state marker for acute depressive episodes. To generalize these findings, future studies on S100B levels as a predictor of antidepressant response should include other antidepressants and patients with other subtypes of major depression as well. These studies will show which treatments might be more effective in patients with low S100B levels. Then, the clinician will be able to choose the right treatment based on this biomarker, reducing patients’ burden of nonresponding.

## Supplementary Material

For supplementary material accompanying this paper, visit http://www.ijnp.oxfordjournals.org/

## Statement of Interest

V.B. is supported by an Erasmus University fellowship and has received funding from the Netherlands Organisation for Scientific Research (NWO, Rubicon incentive). V.A. is a member of advisory boards and/or gave presentations for the following companies: Astra-Zeneca, Eli Lilly, Janssen-Organon, Lundbeck, Otsuka, Servier, and Trommsdorff. T.K.B. receives study support and speaker fees from Lundbeck. The other authors declare that they have no conflicts of interest.

## Supplementary Material

supplementary Figure 1
