# Supplementary material for: S100B Serum Levels Predict Treatment Response in Patients with Melancholic Depression
Source: Int J Neuropsychopharmacol. 2015 Sep 12;19(3):pyv103. doi: 10.1093/ijnp/pyv103 (PMC4815471; doi:10.1093/ijnp/pyv103)
Supplement: supplementary Figure 1 [file pyv103SupplementaryMaterial_correctedOA.docx]

# S100B Serum Levels Predict Treatment Response in Patients with Melancholic Depression

Oliver Ambrée, Veerle Bergink, Laura Grosse, Judith Alferink, Hemmo A. Drexhage, Matthias Rothermundt, Volker Arolt, Tom K. Birkenhäger

Department of Psychiatry, University of Münster, Germany (Dr Ambrée, Ms Grosse, Dr Alferink, Dr Rothermundt, and Dr Arolt); Department of Psychiatry, Erasmus Medical Center Rotterdam, The Netherlands (Drs Bergink and Birkenhäger); Radiology Morphological Solutions, Rotterdam, The Netherlands (Ms Grosse); Department of Immunology, Erasmus Medical Center Rotterdam, The Netherlands (Dr Drexhage); Cluster of Excellence EXC 1003, Cells in Motion, Münster, Germany (Dr Alferink); Department of Psychiatry, Evangelisches Klinikum Niederrhein, Oberhausen, Germany (Dr Rothermundt).

Supplementary Figure 1. Depiction of sample sizes of patient subgroups.

**
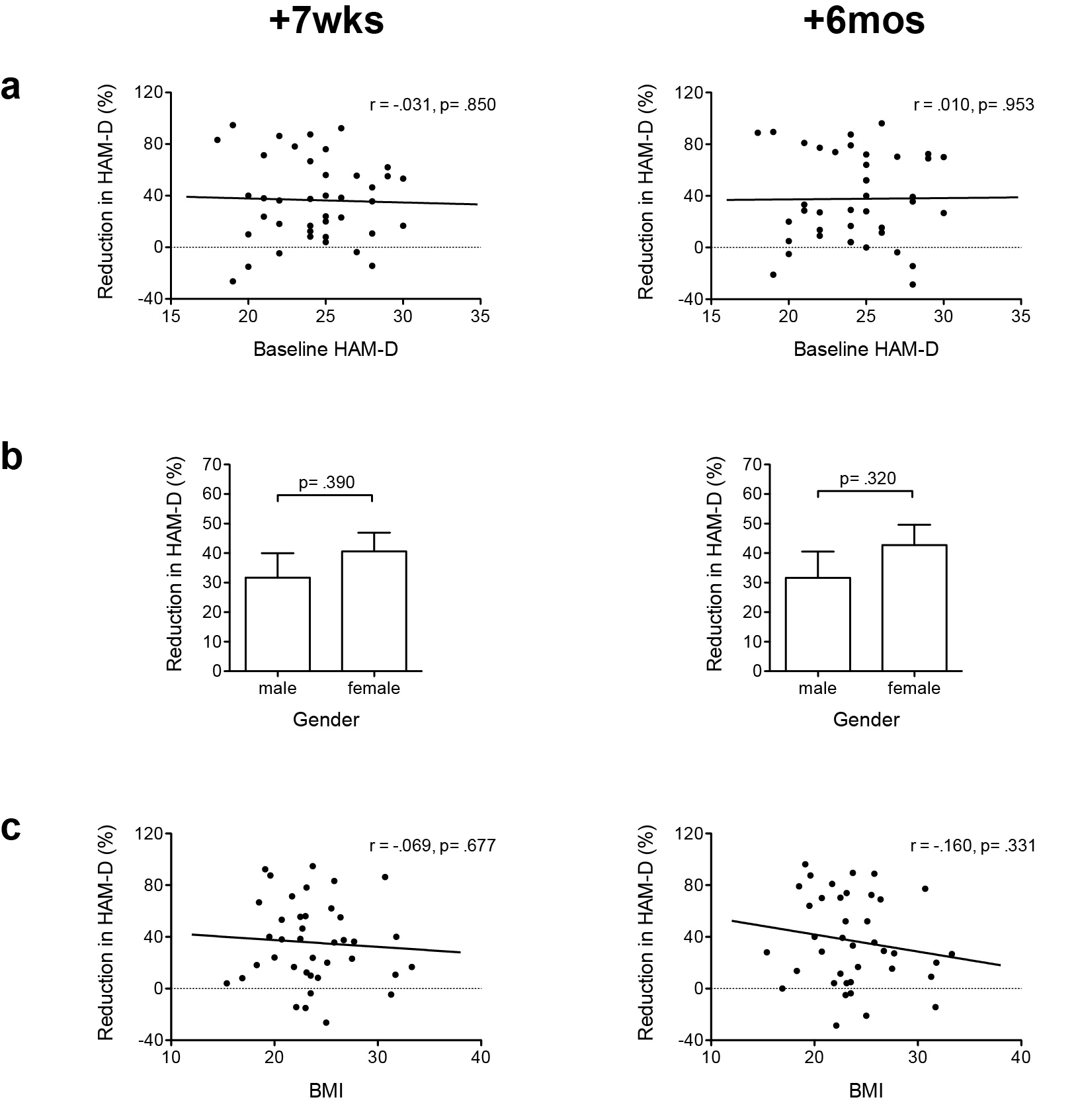
**

Supplementary Figure 2. (a) There was no association between severity of depression as measured by baseline Hamilton Depression Rating Scale (HAM-D) scores and the treatment response as measured as percentage reduction in these scores. (b) Male and female patients did not differ significantly in treatment response. (c) Body mass index (BMI) was also not associated with treatment response.
